# Bidirectional Interplay Between Microglia and Mast Cells

**DOI:** 10.3390/ijms26157556

**Published:** 2025-08-05

**Authors:** Szandra Lakatos, Judit Rosta

**Affiliations:** Department of Physiology, University of Szeged, Dóm tér 10, H-6720 Szeged, Hungary; rosta.judit@med.u-szeged.hu

**Keywords:** microglia, mast cells, cytokines, PAR2, purinergic receptors, histamine, neuroinflammation

## Abstract

Microglia, the brain’s resident innate immune cells, play a fundamental role in maintaining neural homeostasis and mediating responses to injury or infection. Upon activation, microglia undergo morphological and functional changes, including phenotypic switching between pro- and anti-inflammatory types and the release of different inflammatory mediators. These processes contribute to neuroprotection and the pathogenesis of various central nervous system (CNS) disorders. Mast cells, although sparsely located in the brain, exert a significant influence on neuroinflammation through their interactions with microglia. Through degranulation and secretion of different mediators, mast cells disrupt the blood–brain barrier and modulate microglial responses, including alteration of microglial phenotypes. Notably, mast cell-derived factors, such as histamine, interleukins, and tryptase, activate microglia through various pathways including protease-activated receptor 2 and purinergic receptors. These interactions amplify inflammatory cascades via various signaling pathways. Previous studies have revealed an exceedingly complex crosstalk between mast cells and microglia suggesting a bidirectional regulation of CNS immunity, implicating their cooperation in both neurodegenerative progression and repair mechanisms. Here, we review some of the diverse communication pathways involved in this complex interplay. Understanding this crosstalk may offer novel insights into the cellular dynamics of neuroinflammation and highlight potential therapeutic targets for a variety of CNS disorders.

## 1. Introduction

Microglia, glial cells with phagocytic activity in the central nervous system (CNS), play a crucial role in immune reactions in the brain and spinal cord. Microglial cells can change their morphology and activity depending on environmental stimuli. Due to activation, microglia produce both pro- and anti-inflammatory signals and are also involved in repair mechanisms. Microglial activation is therefore involved in the pathogenesis of almost all CNS diseases and their consequent repair mechanisms, including traumatic injuries, stroke, Alzheimer’s disease, multiple sclerosis, Parkinson’s disease, and sensory neuropathies [1]. Extensive research has already revealed several signaling pathways involved in microglial activation, but their therapeutic potential has not been fully exploited to date.

Neuroinflammation, inflammation of the CNS, is implicated in the pathogenesis of several neurological diseases, even if their primary etymology is not associated with immune reactions. Microglial activation plays a prominent role in neuroinflammation. The neuroinflammatory process starts with the release of pro-inflammatory cytokines, chemokines, or reactive oxygen species (ROS) by microglial cells and astrocytes. Microglial activation also manifests itself in reduced expression of tight junction proteins, leading to increased blood–brain barrier (BBB) permeability to neurotoxic substances. These events result in neurotoxicity, synaptic dysfunction, and suppression of neurogenesis [2]. Furthermore, neuroinflammation also involves many other types of immune cells, such as T cells, granulocytes, and mast cells. Mast cells are key effector cells of the immune system, associated with the first line of defense. They can be activated by external pathogens, toxins, environmental antigens, and other signals derived from the innate or adaptive immune system. Mast cells provide immunomodulatory functions through the secretion of several factors that regulate immune responses [3].

In the human brain, mast cells reside on the brain side of the BBB, where they communicate with neurons, astrocytes, microglia, and other brain cells, and in the leptomeninges, where they are located mainly in the perivascular areas that contribute to neuroinflammation by interacting with microglial and endothelial cells [4,5,6].

Pro-inflammatory signals that lead to microglia activation may arise from neurons, blood vessel endothelial cells, or other supporting cells in the brain, such as mast cells. In vitro studies have shown that mast cells can induce microglia activation [7]. Administration of the mast cell degranulator compound 48/80 (C48/80) induced microglial activation and the production of pro-inflammatory factors [8], but microglial activation could not be elicited by C48/80 in mast cell-deficient Kit^w-sh/w-sh^ mice. By contrast, microglial activation and downstream signaling have been shown to be inhibited by the administration of the mast cell stabilizer cromolyn [9,10,11].

Mast cells and microglia may be activated by the same signal, and their function in the regulation of cerebral inflammation is concurrent. However, the unraveling of the communication routes between microglia and mast cells remains to be fully addressed. In this review, we discuss the pertinent interplay between microglia and mast cells and describe the possible bidirectional signaling pathways involved in this interaction.

## 2. Microglia Surveillance

As the resident innate macrophages of the brain, microglia are fundamental in maintaining normal brain functions and neuronal networks by carrying out three main homeostatic functions: sensing, housekeeping, and neuroprotection. The crucial role of microglia in the CNS is demonstrated by the finding that selective elimination of microglia from the brain leads to a marked increase in neuronal death after acute brain injury [1]. Microglia constantly survey their microenvironment and interact with the surrounding elements like neurons, astrocytes, and blood vessels [12,13,14]. Furthermore, microglia are involved in the regulation of neuronal activity, the modulation of synaptic transmission, and the formation, modification, or elimination of synaptic structures. Microglia play an essential role in neurodevelopment by eliminating excess synapses to remove redundant connections through the mechanism of synaptic pruning during postnatal development and throughout adulthood. This promotes the refinement of neural circuits and enhances neuronal network efficiency [15,16,17,18].

**Figure 1:**
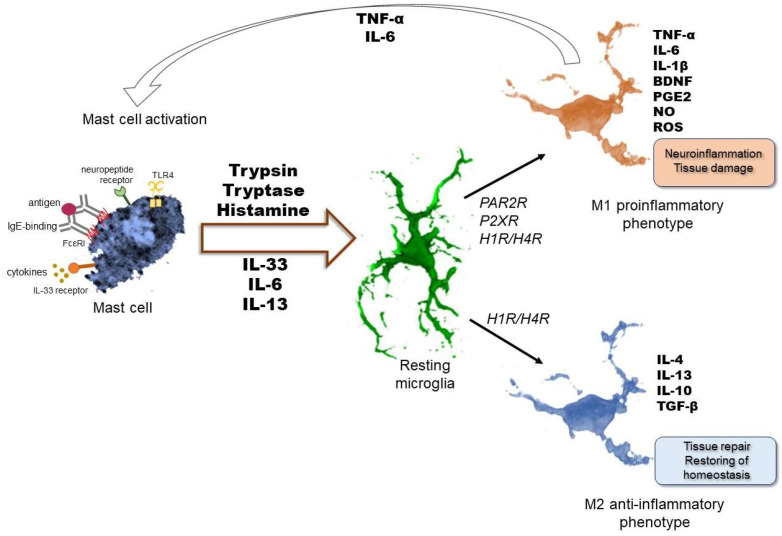
Interactions between mast cells and microglia in the brain. Bidirectional communication may influence not only the release of different mediators but also impact the state of activation of these immune cells. Microglial phenotype switching is influenced by signals arising from their environment. Activated mast cells could be a source of these mediators. Mast cell-derived factors, like different interleukins, trypsin, tryptase, or histamine, trigger the alteration of the microglial phenotype to switch either to M1 pro-inflammatory or M2 anti-inflammatory subclasses. Both the M1 and M2 phenotypes can be characterized by a specific spectrum of pro- or anti-inflammatory mediator release leading to neuroinflammation or tissue repair, respectively. Activated microglia may then affect mast cell activation, resulting in a more prominent inflammatory response. FcεR1: high-affinity immunoglobulin E (IgE) receptor, TLR4: toll-like receptor 4, IL: interleukin, PAR2R: protease-activated receptor 2, P2XR: purinergic P2 receptor, H1R/H4R: histamine receptor 1 and 4, TNF-α: tumor necrosis factor-α, TGF-β: transforming growth factor-β, BDNF: brain-derived neurotrophic factor, PGE2: prostaglandin E2, NO: nitric oxide, ROS: reactive oxygen species.

Microglial activation is a highly complex process triggered by any environmental factor that affects the functional integrity of the nervous system. Various abnormal conditions and signals, such as neurotoxins, neuronal debris, or different types of injuries, can lead to microglia activation characterized by morphological changes, proliferation, migration to the site of injury, phagocytosis, and secretion of inflammatory mediators. Microglial cells in their resting state display a ramified morphology with relatively small cell bodies and long branching processes that constantly survey their surrounding area for extracellular threats by extending and retracting their processes. In response to injury or immune threats, quiescent microglia immediately shift to a reactive form. Microglial activation may be simplified into two polarization states: an M1 pro-inflammatory or reactive phenotype (induced by ‘classical activation’) that initiates an inflammatory response and an M2 phenotype (‘alternative activation’) possessing an anti-inflammatory role [10,19,20,21] (Figure 1); however, this polarization should rather be considered as an M1/M2 differentiation spectrum with several subclasses of polarization. Within injured tissue, microglia exhibit diverse activation states and retain the capability to dynamically shift their functional phenotypes due to inflammatory triggers, transitioning between distinct polarization subclasses [20]. Upon exposure to microbial products such as lipopolysaccharides or infection-associated signals, such as interferons, or in sterile inflammation after trauma, the M1-type microglia can promote inflammatory reactions by secreting pro-inflammatory mediators and neurotoxic molecules. Microglia with the M2 phenotype have high phagocytic activity and contribute to tissue repair mechanisms and the resolution of inflammation by engulfing dead cells. Microglia, especially in the M2 polarized state, also contribute to the maintenance of normal tissue dynamics following injury to achieve tissue homeostasis. Upon activation, Ca^2+^ permeability of cells increases, leading to Ca^2+^ influx, which in turn initiates signaling pathways that eventually cause morphological transformation into a less ramified amoeboid cell with an enlarged cell body and several short and thickened processes [22,23,24]. In addition to morphological changes, microglia activation is also characterized by alterations in gene expression and surface markers, such as the upregulation of the calcium-binding adaptor molecule 1 (Iba1), a calcium-binding microglial protein playing a role in microglial phagocytosis. [23].

Furthermore, microglial activation leads to the production of a wide variety of pro- or anti-inflammatory and cytotoxic factors depending on their activation states (Figure 1). The secretory products include tumor necrosis factor-α (TNF-α), interleukin-6 (IL-6), interleukin-1β (IL-1β), and prostaglandin E2 (PGE2), with high levels of nitric oxide (NO) and ROS resulting in deterioration of surrounding neurons and further activation of microglia [8,9,19,24,25]. The M2 phenotype secretes anti-inflammatory cytokines like IL-4, IL-13, IL-10, and transforming growth factor-β (TGF-β) to balance the pro-inflammatory responses [26]. In addition, microglia have the capacity to modulate their functional and molecular profiles in response to multiple or sequential stimuli, a phenomenon known as microglial priming. Experimental evidence in adult murine models demonstrates that peripheral inflammatory insults can induce long-term changes in microglial reactivity, even after an apparent return to homeostasis between initial and subsequent stimuli. Upon resolution of the initial trigger, microglia retain the potential to be reactivated by a new stimulus or relapse, often resulting in an accelerated or amplified response; however, evidence shows that even their loss of responsiveness may occur, leading to either neuroprotective or deleterious outcomes. This reactivation of microglia is characteristic of chronic or recurring conditions, such as chronic neuroinflammation or neurodegenerative diseases [27,28].

## 3. Mast Cells as Guardians to the Brain

Mast cells are derived from myeloid hematopoietic stem cells in the bone marrow. As early CD34+ progenitors, they circulate in the blood, then differentiate and develop into mature mast cells in the tissues where they reside under the influence of local growth factors [8,9,29,30] such as stem cell factor (SCF) [31,32]. Compared to the skin or the gut, relatively few mast cells reside in the brain, where they are strategically positioned to act as sentries. Despite their sparse representation, brain mast cells seem to be continuously redistributing throughout the brain and can react to different provoking stimuli. Typically, mast cells are located around the third ventricle, choroid plexus, and the leptomeninges, while in the brain parenchyma, they are mainly found in the hippocampus and thalamic hypothalamic region. Mast cells are mostly localized near the neurovascular unit of the BBB on the brain side and in close proximity to microglial cells [9].

Mast cells may be categorized according to several aspects such as the tissue of residence, their granular content, or their surface receptors. There are at least two main subpopulations based on their protease expression: a tryptase-expressing subtype and a tryptase- and chymase-expressing subtype, with a predominant mast cell phenotype of tryptase+ chymase+ [33]. In the brain, the presence of a distinct phenotype has been suggested, expressing chymotrypsin cathepsin G [9,34] and the stem cell factor receptor (c-kit). Furthermore, mature mast cells maintain their plasticity and can shift their phenotype to adapt to local environmental needs. Although mast cells reside in the brain in a very low number, upon their activation they have a significant impact on the permeability of the BBB and the function of the surrounding cells. However, mast cells are really abundant in the brain-covering tissues, the meninges. Substantial evidence has emerged recently that meningeal mast cells have the ability to recruit different types of immune cells to the brain by breaking the integrity of the blood–brain barrier [10,35]. Mast cells can penetrate the BBB and contribute to its disruption in several pathologies, like in age-related neurodegenerative diseases, multiple sclerosis, or hypoxic-ischemic brain damage [36]. The mechanism may involve mast cell-regulated activation of matrix-degrading proteolytic enzymes such as matrix metalloproteinase-2 and -9 (MMP2 and MMP9), or the release of BBB-disrupting factors, such as histamine, which have been found to be key factors in the regulation of BBB permeability [36]. Furthermore, by disrupting the BBB, mast cells also recruit neutrophil granulocytes and T-cells to enter the brain parenchyma [7].

Stimulation of mast cells elicits the secretion of various bioactive mediators via different secretory pathways. Mast cell activation may be achieved through numerous factors such as microbial products through toll-like receptors (TLRs) [37]; immunoglobulin E (IgE) binding; stimulation by different neuropeptides, cytokines, or chemokines; physical activators like temperature or pressure; and cell–cell contact, e.g., through OX40/OX40L [30]. Mast cells express the c-kit receptor for SCF and the high-affinity IgE receptor (FcεR1) for IgE binding [32]; however, it is interesting that under physiological conditions, the brain parenchymal mast cells, unlike the dural mast cells, lack the FcεR1-expression as demonstrated in the mouse hypothalamus. Under pathological conditions, IgE may enter the brain, where it could induce the expression of FcεRI in mast cells [38]. Binding to IgE triggers the activation of the mitogen-activated protein kinase (MAPK) cascade and phosphatidylinositol-specific phospholipase C. MAPK then activates cytokine gene expression to produce inflammatory cytokines in addition to neurosensitizing and vasoactive mediators. In addition to various mediator molecules, mast cells are also able to secrete mitochondrial DNA that acts as an alarmin to induce the secretion of pro-inflammatory cytokines from different immune cells [39].

Mast cell activation leads to rapid release of preformed compounds in minutes. Secretory products include a wide range of substances and their release depends on the type of stimulation. These products include vasoactive amines, such as histamine, gonadotropin-releasing hormone, and serotonin; pro-inflammatory cytokines, like tumor necrosis factor (TNF) and different interleukins (IL-1, IL-18, and IL-33); proteases, such as tryptase, [5,6,29,40], chymase, kininogenases, and carboxypeptidase A3 [32]; arachidonic acid derivatives, including PGE2 and different leukotrienes [33]; and T- and B-cell ligands. Furthermore, a longer-term activation may also be observed due to the de novo synthesis of the mediators mentioned above [23,29,30,32,36]. In addition, different chemokines like chemokine (C-X-C motif) ligand 8 and 10 and chemokine (C-C motif) ligand 2 (CCL2) [41] can also be released later. However, many mediators can be secreted selectively from mast cells without degranulation, for example, vascular endothelial growth factor [41] or IL-6 [39]. Releasing these mediators then triggers other immune effector cell activation in a paracrine manner, or some of these factors may even inhibit further mast cell activation in an autocrine way.

## 4. Key Signaling Pathways Between Microglia and Mast Cells

**Figure 2:**
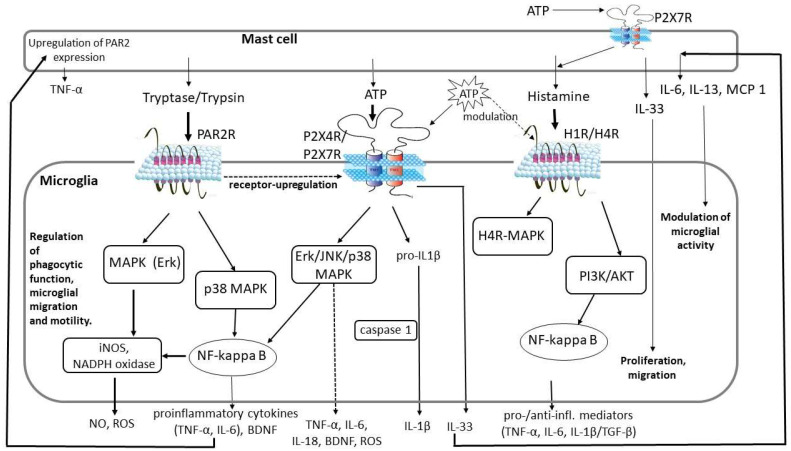
Graphical representation of the main signaling pathways between mast cells and microglia involving PAR2, purinergic, and histaminergic signaling. Activated mast cells release tryptase, which cleaves PAR2R and induces signal transduction pathways via MAPK/Erk and p38 MAPK activation. ATP is a ubiquitous mediator that, in addition to appearing extracellularly in the brain as a result of physiological neuronal activity or neuronal tissue damage, is also released during inflammatory processes by several tissue elements, including mast cells. ATP targets both microglia and mast cells acting through various purinergic receptors such as the cation channel P2X4R or P2X7R. Histamine has a dual action on microglia, inducing the release of both pro- and anti-inflammatory mediators through histamine receptors H1R and H4R. All induced signal transduction pathways regulate microglia phagocytic function and motility. MCP 1: monocyte chemoattractant protein-1, PAR2: protease-activated receptor 2, H1R/H4R: histamine receptor 1 and 4, NO: nitric oxide, iNOS: inducible NO synthase, IL: interleukin, TNF: tumor necrosis factor, BDNF: brain-derived neurotrophic factor, ROS: reactive oxygen species, infl.: inflammatory.

The exact effects of mast cells on microglia and the activation mechanism have not yet been fully understood, but several signaling pathways that are involved in the bidirectional communication between mast cells and microglia have already been outlined (Figure 2). In the following section, we discuss some of these signaling pathways.

### 4.1. PAR2 Signaling

Protease-activated receptor 2 (PAR2) is widely expressed in the central nervous system and is involved in a series of neurodegenerative disorders. Mast cells can activate PAR2 signaling to induce inflammatory reactions. PARs are widely expressed in neurons and glial cells [42], and among PARs, the PAR2 has been found to be activated by trypsin and mast cell tryptase. PAR2 is known to be involved in the pathogenesis of ischemia and neurodegeneration, as well as in eliciting pain hypersensitivity. Furthermore, PAR2 cleavage by mast cell tryptase has been shown to induce microglial activation [40,43,44,45,46]. Mast cell-released tryptase can elicit the release of pro-inflammatory mediators such as TNF-α and IL-6 [47] through the stimulation of microglial PAR2 receptors, the induction of the MAPKs (Erk and p38), and the nuclear factor kappa-light-chain-enhancer of activated B cells (NF-κB) signaling pathway [40,45,48]; this action leads to the upregulation of P2X4 purinergic receptor [47]. Microglial activation also promotes the release of NO and ROS through the NADPH oxidase, myeloperoxidase, and inducible NO synthase activated through the MAPK/ERK and NF-κB pathways [49]. The pro-inflammatory mediator IL-6 released from microglia plays a fundamental role in CNS inflammation-related responses such as neurogenesis, gliogenesis, cell growth, cell survival, myelination, and demyelination. Following brain injury, IL-6 expression levels are elevated in cerebral spinal fluid and brain homogenates [40]. In turn, microglia-derived IL-6 and TNF-α may upregulate PAR2 expression in mast cells, resulting in mast cell activation and the release of TNF-α [47]. Furthermore, mast cell tryptase via PAR2 receptor triggers the release of brain-derived neurotrophic factor (BDNF) [5,30,44], and also controls microglial chemotaxis [50].

### 4.2. Purinergic Signaling

ATP as an intracellular messenger mediates cell–cell signaling in a variety of tissues, including the central nervous system, where it has an important role in eliciting functional responses of mast cells and microglial cells [5]. ATP is a ubiquitous mediator; therefore, it is synthesized by all types of metabolically active cells of the nervous system, involving nerve terminals, astrocytes, endothelial cells, and microglial cells themselves as well [24]. In addition to ATP appearing extracellularly in the brain as a result of physiological neuronal activity, acting as a danger signal, it is also released or leaked by injured cells as a consequence of pathological and/or damage-related events [51]. One of the most important mechanisms involves purinergic P2 receptors. The P2 receptor (P2R) family comprises two subfamilies: the ligand ATP-gated ionotropic P2X receptors and the G protein-coupled metabotropic P2Y receptors.

Four members of the P2XRs, the P2X1, P2X4, P2X6, and P2X7, have been found to be expressed in mast cells modulating their activities, such as their Ca2+ signal and degranulation. Mast cell degranulation has been found to be exclusively induced through the P2X7 pathway among P2X channels [5]. P2X7 activation in mast cells leads to the release of histamine, which stimulates microglia to release TNF-α, IL-1β, and IL-6 [5]. Microglia also abundantly express P2X4 in addition to P2X7 receptors [51,52]. Microglial cells expressing P2X7 are found to be evenly distributed in the forebrain, while P2X4-expressing cells are localized around the blood vessels and in the subarachnoid space [52]. The expression of P2X receptor subunits dynamically changes during embryonic and postnatal development, resulting in the final expression of mRNAs for only the P2X4 and P2X7 subunits whose expression pattern may be altered in various neurological diseases, leading to the upregulation of other P2X subunits [52].

P2 receptor activation in microglia by adenosine 5′-triphosphate (ATP), for instance, leads to the release of IL-33, which can trigger the release of IL-6, IL-13 [30], and monocyte chemoattractant protein 1 (MCP 1) [53] from mast cells. Then, these cytokines in turn may influence microglia activity [30]. IL-13 has been found to effectively trigger the switch of the microglial phenotype from the M1 to M2 state, and can reduce the production of inflammatory mediators [54]. The microglial response to IL-6 may be involved in complement-mediated tissue damage and neuronal synapse destruction, as binding to IL-6 leads to the production of complement C3 protein by microglia [55]. MCP-1 is a key chemokine that influences microglial movement. It can induce proliferation of isolated microglia; however, it appears to not be involved in the regulation of the production of pro-inflammatory cytokines or in the induction of morphological changes in microglia [56].

The connection between the P2X7 receptor and IL-1β signaling was first described in microglia in which the activation of the P2X7 receptor leads to the cleavage of pro-IL-1β by caspase 1 [24,57]. In addition to the release of IL-1β, the activation of microglial P2X7 receptors by mast cell-derived ATP leads to the release of several other mediators such as BDNF and TNF-α via the Erk/JNK/p38 MAPK signaling pathway, CCL2, IL-6, IL-18 [5,24,51], and microglial response factor-1 (MRF-1) [24], as well as the release of activated oxygen species and induction of proliferation [57]. Interestingly, activation of P2 receptors is also required for the rapid degradation of ATP by microglial ectonucleotidase enzymes, which is crucial in keeping microglial basal phagocytic activity at a low level and maintaining microglial ramified morphology [51].

As mentioned earlier, experimental data have shown that purinergic signaling could also be mediated by the tryptase-induced PAR2 signaling. Co-cultured with activated mast cells, microglia display an elevated level of P2X4 receptor expression promoted by the activation of PAR2 [44].

### 4.3. Histamine Signaling

Histamine has been found to be crucial in the modulation of microglia-mediated neuroinflammation. In the brain, mast cells are the main non-neuronal source of histamine that can be released upon degranulation [58]; however, it was shown that microglia can also produce histamine [59]. Mast cell-derived histamine stimulates microglial cell motility and also triggers the secretion of both pro-inflammatory cytokines, like TNF-α, IL-6, and IL-1β [60,61,62], and anti-inflammatory cytokines, like TGF-β [63] and IL-10 [64]. It has been demonstrated that microglia constitutively express all four histamine receptors (H1R, H2R, H3R, and H4R); however, only H1R and H4R antagonists lead to reduced histamine-induced TNF-α and IL-6 production and to a less-activated MAPK and PI3K/AKT signaling pathway [36]. This suggests that among all histamine receptors, histamine mainly acts via H1R and H4R [23,65,66,67].

Microglia activation and their phagocytic function may be mediated by histamine released from activated mast cells [23]. Histamine-induced microglial phagocytosis is modulated by ATP, which at higher concentrations appears to have an inhibitory effect on phagocytosis, as intense mast cell degranulation resulting in high ATP levels reduced the phagocytosis rate [23]. Mast cells have been suggested to be involved not exclusively in the activation of microglia but also in their normal functioning as well. It was found that in histamine deficiency knock-out mice, the number of microglia was not changed, but their morphology showed reduced ramifications and decreased expression of insulin-like growth factor-1 and of H4 receptor [67].

## 5. Other Routes for the Activation of Mast Cells and Microglia

### 5.1. Toll-like Receptors

Toll-like receptors play a fundamental role in the innate immune system as one of the large receptor families of pattern-recognition receptors (PRRs) that enable the sentinel cells, such as microglia and mast cells, to recognize conserved pathogen motifs. In addition to sensing microbes, these PRRs are also involved in the detection of ‘danger’ signals from damaged cells. For recognizing microbes and tissue damage, other receptors of the PRR family may be present in different immune cells such as nucleotide-binding and oligomerization domain (NOD)-like receptors, scavenger receptors, or C-type lectin receptors [9].

Among the known TLRs, in mast cells and microglia, TLR2 and TLR4 are of main importance. Activation of TLR2/TLR4 in mast cells results in cytokine release leading to microglia activation and the recruitment of immune cells to the site of injury. In addition, TLR2/TLR4 signaling in mast cells also upregulates several chemokines, such as CCL5/RANTES, which can also induce a pro-inflammatory profile in microglia. In turn, microglia-derived IL-6 and CCL5 may modulate the surface expression of TLR2/TLR4 in mast cells [68].

Activation of TLRs may be achieved both exo- and endogenously through binding to pathogen-associated molecular patterns (PAMPs) or danger-associated molecular patterns (DAMPs), respectively. In the CNS, similar activation may be induced by neurodegeneration-associated molecular patterns (NAMPs) originating from a wide range of stimuli; however, the exact identities of the NAMPs are still to be clarified. NAMPs may include constituents of dying neurons (like ATP and mitochondrial DNA), heat shock proteins, and deposits arising from neurodegenerative disorders, such as β-amyloid or α-synuclein [9,69,70].

TLR activation depends on receptor dimerization and recruitment of the adapter proteins myeloid differentiation primary response protein 88 (MyD88) or Tir-domain-containing adaptor-inducing interferon-β (TRIF). These adaptor proteins mediate downstream signaling cascades leading to the activation of transcription factors, including NF-κB [70,71,72]. Finally, transcription factors induce the generation of inflammatory mediators, like interleukins (IL-1β and IL-6), TNF-α, chemokines (macrophage inflammatory protein-1 and MCP-1), acute-phase proteins, and oxidative stress-related enzymes contributing to neuroinflammation [9,69,70].

Table 1 summarizes the above-mentioned signaling pathways and their roles in the interaction between microglia and mast cells.

### 5.2. Involvement of Inflammasomes

Both mast cells and microglia possess key cytosolic multiprotein complexes, called inflammasomes, which can detect and eliminate diverse stimuli, including microbial and host- and environment-derived triggers, like PAMPs, DAMPs, and NAMPs [73]. The best known inflammasome is the NOD-, LRR-, and pyrin domain-containing protein 3 (NLRP3) inflammasome, which consists of three main components: the NLR sensor protein; the Apoptosis-associated Speck-like protein containing a caspase-activating and recruitment domain (ASC) adaptor; and the effector protease, caspase-1 [74,75]. Recently, NLRP3 has been shown to be required in the process of mast cell degranulation [76]. NLRP3 activation and assembly depend on dual stimulation, starting with a priming signal delivered by TLR-stimulation. The second signal is the activating signal that can be triggered by a variety of stimuli, like NAMPs, exogenous ATP, reactive oxygen species, certain bacterial toxins, or lysosomal proteases [49,74]. This activator signal leads to the construction of the active form of the inflammasome that is capable of activating caspase-1 [77]. Caspase-1 is a key component in the secretion of IL-1β and IL-18 by proteolytic cleavage of pro-IL-1β and pro-IL-18 [72,73]. Increased expression of IL-1β and IL-18 is often detected in CNS infection, brain injury, and neurodegenerative diseases, and has an important role in inflammatory cascades [49]. Therefore, NLRP3 has been reported to be a promising therapeutic target in neurological diseases associated with neuroinflammation [78].

## 6. Mast Cell-Related Signaling in M1/M2 Polarization

As discussed above, activated microglia acquire different polarization states such as M1 ‘pro-inflammatory’ and M2 ‘anti-inflammatory’ phenotypes, and they can switch from one state to another. The M1 reactive state of microglia is induced by interferon-γ and lipopolysaccharide, a process known as ‘classical activation’ [79], whereas anti-inflammatory cytokines such as TGF-β, IL-4, IL-10, or IL-13 typically keep microglia in the M2 state [79]. However, dynamic transformation among reactive microglial states has been revealed in neurodegenerative disorders or following neuronal injuries. Several regulators and signaling pathways are involved in the microglial phenotype switch between the M1 and M2 phenotypes [80]. However, this binary concept of microglia classification (M1/M2) does not cover the entire spectrum of microglial activation states; it can be used to distinguish between neuroprotective and neurotoxic microglial function in the pathobiology of CNS disorders.

### 6.1. Microglia Polarization: From M1 to M2 or from M2 to M1

Several lines of evidence indicate the polarization of microglia to the M1 phenotype in the pathogenesis of neurodegenerative diseases such as Alzheimer’s disease, Parkinson’s disease, or amyotrophic lateral sclerosis (ALS) [81,82,83]. Basically, oxidative stress, which is typically associated with neurodegenerative disorders, and the consequent increase in ROS level can induce a phenotypic shift from M2 to M1 [84]. In Parkinson’s disease, the accumulated α-synuclein seems to directly induce M1 activation [85]. According to extensive genetic studies, the superoxide dismutase (mSOD1) has been suggested to possess a key role in the phenotypic shift of microglia toward the M1 type in ALS [86,87].

Experimental studies have also shown polarization of M1 microglia toward the M2 phenotype following neuronal injuries. PPAR-γ, JAK/STAT6, and TLR2/IRAK1/NF-κB pathways are involved in this polarization process (M1 to M2) [88,89]. M2 polarization is typically induced by anti-inflammatory cytokines such as IL-4 and IL-13 [79,90]. These cytokines activate the STAT6 and PPAR-γ signaling pathways. Recent evidence has indicated that the GAS6/AXL signaling pathway, which is critical in cell survival, proliferation, or migration [91], induces the expression of anti-inflammatory genes and also increases phagocytic function by upregulating certain receptors related to phagocytosis [92,93]. The transcription factor NF-κB has been identified as a key regulator in the signalization to switch microglia from the M1 to M2 phenotype, as its inhibition induces an increase in the M2 microglia ratio due to a decrease in the M1 phenotype [94].

### 6.2. Mast Cell-Induced Microglial Polarization

Mast cell-induced signaling pathways are supposed to be involved in microglial M1/M2 polarization processes. Based on the related findings, we assume that mast cell activation may promote phenotypic switching of microglial cells. Mast cells are basically thought to initiate immune responses; however, several lines of evidence indicate that mast cells also have a potential to reduce inflammation and help in tissue remodeling. Therefore, it deserves to be discussed whether mast cell-induced signaling drives microglia towards a pro-inflammatory or anti-inflammatory phenotype.

As discussed above, microglia are driven towards the M2 phenotype typically by anti-inflammatory cytokines such as TGF-β, IL-4, IL-10, and IL-13. Mature mast cells can produce a high amount of TGF-β [95], a key regulator of microglia function. IL-4 and IL-13 are also synthesized and released by mast cells upon IgE-mediated stimulation [96]. Therefore, mast cell activation appears to be able to promote the polarization from M1 to M2.

Mast cell-derived signaling basically seems to induce M1 activation. Mast cells are the most relevant source of histamine, a key mediator of adaptive and innate immune responses. Histamine antagonists basically promote anti-inflammatory processes targeting histamine receptors 1–4 (H1R-H4R). For example, the H1 receptor antagonist Clemastine reduces the reactive state of microglia and modulates the expression of microglia-related inflammatory genes [97]. Inhibition of H3R also provides a neuroprotective action, as it promotes the shift from the M1 to the M2 phenotype [98].

Adenosine is released from several different sources during inflammation, including mast cells that also secrete a large amount of ATP upon activation [5,99]. The classical way of activation (M1) is typically induced via receptors involved in innate immune responses, such as pattern recognition receptors and NOD-like receptors [20]. P2X and P2Y purinergic receptors are involved in microglial activation and motility. Janks et al. proved that the ATP receptor P2X7 has a key role in permeabilization and activation of microglia [100]. However, an ATP-sensitive potassium channel, Kir6.1/K-ATP, has been shown to promote M2 polarization of microglia in a mouse model of Parkinson’s disease [101]. In addition, a purinergic receptor, P2Y12, seems to be related to the resting state of microglia, as its expression is highly abundant in the resting state, but reduced upon activation [102].

Polarization from the M1 to M2 phenotype results in a reduction in reactive microglia and provides a neuroprotective effect; therefore, M1/M2 shift has long been considered as a potential therapeutic strategy in neurodegenerative disorders. Inhibition of M1 activation by anti-inflammatory drugs like cyclooxygenase inhibitors, aspirin, or acetaminophen was not effective in neurodegenerative models [103,104]. A promising neuroprotective strategy might require the reversal of the M1 phenotype simultaneously with the promotion of M2 activation [105]. Administration of a purinergic receptor P2X7 antagonist has been shown to provide a neuroprotective effect by reducing microgliosis. P2X7 antagonism was associated with reduced M1 markers and increased expression of M2 markers [106]. Inhibition of H3R also provides a neuroprotective action by promoting polarization from M1 to M2 [98].

According to current studies, the role of either histamine or ATP signaling in microglia polarization is more controversial and cannot be clearly defined according to the bipolar M1/M2 system. Taken together, mast cell-induced signaling in microglial polarization should be considered in the process of neuroinflammation. Further studies are needed to discriminate the effect of single pathways and to clarify the effect of mast cell activation on microglial polarization.

## 7. Conclusions

In this review, we introduced the interaction of microglia and mast cells during inflammatory processes and focused on the pathways involved in bidirectional signaling. Emerging evidence indicates that reciprocal activation loops between mast cells and microglia can amplify inflammatory reactions by enhancing the release of pro-inflammatory mediators. Bidirectional communication concerns several signal transduction pathways, especially the tryptase, purinergic, and histamine signaling pathways, thereby suggesting its substantial role in inflammatory processes of the central nervous system. Based on relevant research data, dynamic interaction between microglia and mast cells may be implicated in various CNS pathologies, including traumatic brain injury, stroke, and various neurodegenerative disorders, like multiple sclerosis or Alzheimer’s disease. Understanding this interplay may provide deeper insights into microglial activation and may open new insights for neuroprotective therapies. However, further studies are needed to clarify the role of single mast cell-induced pathways in M1/M2 microglia polarization. For example, the effects of reactive microglia on mast cells have not been widely studied until now. It has been demonstrated that microglia-derived factors such as IL-6 or TNF-α may modulate mast cell functions by modifying expression of TLRs or PAR2 receptors; however, more extensive research is required to understand the impact of these interactions. We conclude that studying the bidirectional interplay between mast cells and microglia might give valuable information to explore new targets in neuroprotective therapies.

## Figures and Tables

**Table 1:** Summary of the main mast cell-induced microglial activation pathways.

Factors Released from Activated Mast Cells	Microglial Receptor	Effect on Microglia	Role
Trypsin, tryptase	PAR2 receptor	Release of TNF-α, IL-6, NO, ROS, BDNF. Upregulation of P2X4 receptor.	Microglia-derived IL-6 and TNF-α: upregulation of PAR2 expression in mast cells resulting in mast cell activation and TNF-α release. IL-6: neurogenesis, gliogenesis, cell survival, myelination/demyelination.
ATP	P2X4/7 receptor	Release of TNF-α, IL-6, IL-1β, IL-18, CCL2, MRF-1, ROS. Release of IL-33 from preactivated microglia.	IL-33 induces the secretion of IL-6, IL-13, and MCP1 from mast cells, which could affect microglial activity. Microglia migration to the site of injury. Proliferation, phagocytosis. Degradation of ATP by microglial ectonucleotidases.
Histamine	H1 and H4 receptor	Release of both pro- (TNF-α, IL-6, IL-1β) and anti-inflammatory (TGF-β, IL-10) cytokines.	Regulation of microglial migration and motility. Microglial phagocytosis (modulated by ATP). Normal microglial functioning.
PAMP/DAMP/NAMP	TLR2/TLR4	The release of IL-6 and CCL5 modulates TLR2/4 expression of mast cells.	Cytokine/chemokine release, which induces pro-inflammatory response in microglia and the recruitment of immune cells to the site of injury.

## Abbreviations

The following abbreviations are used in this manuscript:

ALS	amyotrophic lateral sclerosis
BBB	blood–brain barrier
C48/80	compound 48/80
CCL2	chemokine (C-C motif) ligand 2
CNS	central nervous system
Iba1	calcium-binding adaptor molecule 1
IgE	immunoglobulin E
IL	interleukin
MAPK	mitogen-activated protein kinase
MMP	matrix metalloproteinase
MRF-1	microglial response factor-1
NF-κB	nuclear factor kappa-light-chain-enhancer of activated B cells
NO	nitric oxide
NOD	nucleotide-binding and oligomerization domain
NLRP3	NOD-, LRR-, and pyrin domain-containing protein 3
PAR	protease-activated receptor
PGE2	prostaglandin E2
ROS	reactive oxygen species
SCF	stem cell factor
TGF-β	transforming growth factor β
TLR	toll-like receptor
TNF	tumor necrosis factor
